# Cathepsin S contributes to influenza-induced lung injury by driving inflammation, promoting apoptosis, and disrupting epithelial barrier integrity

**DOI:** 10.1128/spectrum.01128-25

**Published:** 2025-11-19

**Authors:** Tianxin Ma, Chunguang Yang, Yang Wang, Chuanmeizi Tu, Jiawei Zhang, Kailin Mai, Shengzhen Wu, Hongxuan Zhou, Shengfeng Li, Sulan Ye, Lixi Liang, Qingsheng Huang, Zhenhui Zhang, Zhengshi Lin, Weiqi Pan, Zifeng Yang

**Affiliations:** 1 State Key Laboratory of Respiratory Disease, National Clinical Research Center for Respiratory Disease, Guangzhou Institute of Respiratory Health, The First Affiliated Hospital of Guangzhou Medical University, Guangzhou, China; 2 Guangzhou National Laboratory, Guangzhou, China; 3 Respiratory Disease AI Laboratory on Epidemic and Medical Big Data Instrument Applications, Faculty of Innovation Engineering, Macau University of Science and Technology, Taipa, Macau SAR, China; 4 State Key Laboratory of Quality Research in Chinese Medicine, Macau Institute for Applied Research in Medicine and Health, Macau University of Science and Technology, Taipa, Macau SAR, China; 5 Department of Critical Care, The Second Affiliated Hospital of Guangzhou Medical University, Guangzhou, China; Shandong First Medical University, Jinan, Shandong, China

**Keywords:** cathepsin S, inflammatory response, apoptosis, epithelial barrier

## Abstract

Influenza virus infection causes significant morbidity and mortality worldwide, with severe cases often driven by excessive host inflammatory responses and disruption of epithelial barrier function. Here, we identified cathepsin S (CTSS), a lysosomal cysteine protease, as a key mediator of influenza-induced lung injury. Influenza virus infection upregulated CTSS in a time-dependent and dose-dependent manner, leading to lysosomal membrane permeabilization and cytoplasmic release of CTSS, which correlated with increased apoptosis and loss of epithelial barrier integrity. Knockdown of CTSS reduced proinflammatory cytokine production, apoptosis, and barrier disruption in A549 cells. Air–liquid interface airway epithelial cultures further validated the essential role of CTSS in preserving epithelial barrier integrity. *In vivo*, pharmacological inhibition of CTSS alleviated lung inflammation and disease severity in infected mice without reducing viral titers. Tumor necrosis factor-alpha (TNF-α) strongly induced CTSS activation, but failed to restore apoptosis and inflammatory responses in CTSS-knockdown cells, suggesting that CTSS acts downstream of cytokine signaling. These findings reveal a central role for CTSS in linking viral infection, cytokine storm, and epithelial damage and highlight CTSS as a promising target for host-directed therapy against severe influenza.

**IMPORTANCE:**

Severe influenza is often driven by excessive host inflammation and epithelial barrier disruption, yet the molecular mediators connecting viral infection to these pathological processes remain poorly defined. This study identifies cathepsin S (CTSS) as a central driver of influenza-induced epithelial injury, acting downstream of viral replication and cytokine signaling to promote apoptosis, inflammation, and barrier disruption. Using both *in vitro* and *in vivo* models, we demonstrate that inhibiting CTSS preserves barrier integrity and attenuates inflammation despite having no beneficial effect on viral clearance. These findings provide mechanistic insight into influenza pathogenesis and support CTSS as a promising target for host-directed interventions to mitigate cytokine storm-driven lung injury in severe respiratory viral infections.

## INTRODUCTION

Influenza is an acute, highly contagious respiratory disease caused by influenza viruses that occur annually and occasionally cause pandemics (1). Globally, influenza is estimated to cause approximately 1 billion infections each year, including 3–5 million cases of severe illness and 290,000–650,000 respiratory-related deaths (2, 3). Typical clinical features include the sudden onset of fever, cough, sore throat, myalgia, and fatigue (4, 5). While most cases are self-limiting, the disease can rapidly progress to severe complications in vulnerable populations, including acute lung injury and, in critical cases, acute respiratory distress syndrome with multi-organ dysfunction, often leading to death (6, 7).

Influenza virus primarily infects the respiratory tract and replicates in epithelial cells. Alveolar epithelial cells are the key components of the air-blood barrier, which consists of alveolar epithelium, basement membrane, and endothelial cells. During acute lung injury, this barrier is disrupted by damage to intercellular junctions as well as injury and death of epithelial and endothelial cells (8). The extracellular matrix (ECM), a major component of the cellular microenvironment, also contributes to influenza-induced lung injury through extensive remodeling and degradation (9, 10).

Most mechanistic studies have focused on immune cell responses and inflammatory signaling, but there remains a significant gap in understanding the direct pathogenesis of influenza-induced acute lung injury. Cathepsin S (CTSS) has recently garnered attention for its role in inflammation and infection (11–13). CTSS is a lysosomal cysteine protease with structural features that distinguish it from other family members such as cathepsin B (CTSB) and cathepsin L (CTSL) (14). Importantly, CTSS maintains enzymatic activity across a broad pH range, with high activity in both acidic and neutral environments. Its substrates include multiple ECM components—laminin, fibronectin, elastin, and collagen—as well as basement membrane constituents such as chondroitin sulfate, heparan sulfate, and proteoglycans (15). Researchers have enriched their understanding of this protease and found that it is involved in the regulation of a series of key physiological processes, including cell cycle regulation, tissue remodeling, inflammation, and immune response (16). Pathologically, while the role of CTSS in antigen presentation has long been recognized, CTSS has been implicated in the pathogenesis of some viral infections (17). CTSS is primarily produced by antigen-presenting cells such as macrophages but can also be expressed and secreted by airway epithelial cells, alveolar epithelial cells, and vascular endothelial cells (18, 19).

This study aimed to investigate the role of CTSS in the pathogenesis of influenza infection, with a particular focus on acute lung injury. Understanding the contribution of CTSS to influenza pathology may provide new insights and support the development of host-directed therapeutic strategies.

## MATERIALS AND METHODS

### Cells and virus

MDCK (NBL-2) cells (American Type Culture Collection [ATCC], CCL-34) were cultured in minimum essential medium (Gibco) supplemented with 10% fetal bovine serum (FBS; Gibco) at 37°C with 5% CO_2_. A549 cells (ATCC, CCL-185) were cultured in Dulbecco’s modified Eagle medium/F12 (1:1) (DMEM/F12, Gibco) supplemented with 10% FBS, under the same conditions. The Influenza A/Puerto Rico/8/1934 (H1N1) (PR8) virus (ATCC, VR-95PQ) was propagated in 9–11-day-old embryonated chicken eggs and titrated in MDCK cells using either plaque assays or 50% tissue culture infective dose (TCID_50_) assays (20, 21).

### Animal studies

Specific pathogen-free, 6–8-week-old female C57BL/6J mice were obtained from Beijing Vital River Laboratory Animal Technology Co., Ltd. and housed in individually ventilated cages. In accordance with ethical guidelines, any animal that lost more than 25% of its body weight was humanely euthanized.

For influenza infection, mice were anesthetized with isoflurane and intranasally inoculated with various doses of PR8 virus. As a control, one group of mice received an equal volume of phosphate buffered saline (PBS). The body weight and survival rate were monitored for 14 days post-infection (dpi). Lungs and bronchoalveolar lavage fluid (BALF) were collected at designated time points for viral titration, pathological examination, CTSS activity, and transcriptomic analysis.

For CTSS inhibition, mice received intraperitoneal injections of 30 mg/kg LY3000328 (MedChem Express, USA, HY-15533) or vehicle twice daily for 5 days. Mice were inoculated intranasally with 100 PFU of PR8 virus 4 h after the first dose of LY3000328. The body weight and survival rate were monitored for 14 dpi, and lungs from infected mice were harvested at 5 dpi for viral titration, histopathology, CTSS activity, and cytokine expression analysis.

### Transcriptome analysis

C57BL/6J mice were infected with specified doses of the virus, with mock-infected mice serving as controls. Lung tissues were harvested at designated time points, and total RNA was isolated using TRIzol (Invitrogen, USA, 15596026CN) according to the manufacturer’s instructions. The RNA was quantified, and libraries were prepared at Novogene Bioinformatics Technology Co., Ltd. (Beijing, China). Libraries were sequenced on the NovaSeq 6000 platform (Illumina, USA) using 150 bp paired-end reads. Raw reads were processed and trimmed to ensure high-quality, reliable data, which were then mapped to the mouse genome using HISAT2 (v2.0.5). Gene expression levels were quantified as fragments per kilobase of transcript per million mapped reads (FPKM) using FeatureCounts (v1.5.0-p3). Differentially expressed genes (DEGs) were identified using DESeq2 (v1.20.0), with a significance threshold of *P* adj ≤ 0.05 and |log_₂_(fold change)| ≥ 1.

Functional enrichment analysis of DEGs was performed in R (v4.2.2) using clusterProfiler, enrichplot, GOplot, ggplot2, and the org.Mm.eg.db database. GO and KEGG pathway analyses used *P* value and *q* value cutoffs of 0.05. ECM-related genes were curated based on the Gene Ontology database.

Weighted gene co-expression network analysis (WGCNA) identified clusters of co-regulated genes, or “modules,” associated with specific infection parameters. Using unsupervised hierarchical clustering of FPKM data for genes expressed above a threshold of 0.5 across all time points, a weighted adjacency matrix was constructed with a soft threshold power of 14 to approximate a scale-free topology. Thirty modules were identified by average linkage hierarchical clustering. Module-trait relationships, membership, and gene significance were assessed using Pearson correlation. Modules significantly correlated with traits such as time post-infection, infection status, weight change, and viral titer (*P* value < 0.05) were further analyzed. Hub genes were identified using Cytoscape (v3.9.1) based on the topological overlap matrix of the relevant module.

### CTSS knockdown in A549 cells

CTSS-targeting siRNAs [siCTSS #1: 5′-CAUGUUCAAAGUACACUGA (dT)(dT)-3′ and siCTSS #2: 5′-CAAUGGGAAUGCACUCAUA (dT)(dT)-3′] and a scramble control siRNA [siSCR: 5′-UUCUCCGAACGUGUCACGU (dT)(dT)3′] were purchased from Tsingke Biotechnology (Beijing, China). Transfections were performed with Lipofectamine RNAiMAX (Thermo Fisher Scientific, USA, 13778075) following the manufacturer’s instructions. Final siRNA concentration was 100 nM in six-well plates.

### Influenza infection in A549 cells

A549 cells were seeded 12 h before transfection and infected with the influenza virus at the indicated multiplicity of infection (MOI) 24 hours post-transfection. After a 1.5 h absorption, the viral inoculum was removed, and the wells were replenished with DMEM/F12 supplemented with 1 µg/mL tosyl phenylalanyl chloromethyl ketone-treated trypsin (Sigma, USA, 4370285).

For the extraction of lysosomes, supernatant, lysosomes, and cytoplasm were isolated at the indicated time points post-infection using the Lysosome Protein Isolation Kit (BestBio, China, BB-31452), following the manufacturer’s instructions.

For the recombinant protein treatment experiment, human-CTSS (MedChem Express, USA, HY-P7756) was added at the indicated doses 1.5 hours post-infection (hpi), cells were harvested and lysed 24 hpi for subsequent experiments.

For TNF-α treatment, human TNF-α protein (MedChem Express, USA HY-P7058) was added at 100 ng/mL 1.5 hpi. Cells were harvested and lysed at 24 hpi for subsequent analyses.

Gene expression was measured by real-time quantitative polymerase chain reaction (RT-qPCR), protein levels by western blotting, and enzymatic activity by CTSS activity assays at designated time points.

### Real-time qPCR

Total RNA from tissues or cells was extracted with TRIzol reagent (Invitrogen, USA, 15596026CN). cDNA was synthesized using HiScript IV RT SuperMix (Vazyme, China, R423). Real-time qPCR was performed with Universal SYBR qPCR Master Mix (Vazyme, China, Q712) according to the manufacturer’s instructions. Primer sequences are listed in Table S1.

### Western blotting

Lung or cell lysates were prepared in radio immunoprecipitation assay (RIPA) lysis buffer (Beyotime, China, P0013) supplemented with protease inhibitor phenylmethanesulfonyl fluoride (PMSF). The lysates were mixed with 5× SDS loading buffer and denatured at 95°C for 10 min. Samples were then separated by SDS-PAGE and transferred to hydrophobic PVDF membranes (Millipore, USA, IPVH00010). The membranes were blocked with 5% skim milk for 1 h at room temperature, then incubated with primary and horseradish peroxidase (HRP)-conjugated secondary antibodies. The primary antibodies used included anti-human CTSS rabbit polyclonal antibody (Proteintech, China, 27538-1-AP), anti-mouse CTSS rabbit monoclonal antibody (Affinity, UK, DF8246), anti-β-actin rabbit monoclonal antibody (Abcam, UK, ab8227), anti-PARP1 monoclonal antibody (Gene Tex, USA, GTX100573), anti-GSDMD-N monoclonal antibody (Abcam, UK, ab215203), anti-pMLKL monoclonal antibody (Abcam, UK, ab187091), anti-E-cadherin antibody (Abcam, UK, ab40772), anti-cleaved-PARP1 rabbit monoclonal antibody (Zenbio, China, R380374), anti-β-tubulin rabbit monoclonal antibody (Zenbio, China, R380628), anti-influenza A virus NP monoclonal antibody (Sino Biological, USA, 11675-T62), and anti-LAMP1 monoclonal antibody (CST, USA, 9091S). Detection was performed using HRP-conjugated goat anti-rabbit IgG (H&L) (Abcam, UK, ab6721).

Band intensities were quantified using ImageJ. Target proteins were normalized to loading controls, and data are presented as fold change relative to controls.

### CTSS activity assay

CTSS activity was measured with the CTSS Activity Assay Kit (Abcam, UK, ab65307) following the manufacturer’s protocol. Briefly, 50–200 μg of tissue extracts, cell lysates, or BALF were prepared in lysis buffer, and the supernatants were collected. Protein concentrations were determined using the BCA Protein Assay Kit (Beyotime, China, P0009). For activity measurements, 50 µL sample, 50 µL of reaction buffer, and 2 µL of substrate were mixed in a 96-well white plate and incubated at 37°C for 1–2 h. Fluorescence was recorded using 400 nm excitation and 505 nm emission.

### Cell death detection

A549 cells were seeded in 96-well plates, then the siRNA transfection and influenza virus infection were carried out in sequence according to the method described earlier. At 24 hpi, supernatants were collected and used for cell death detection using the Cytotoxicity LDH Assay Kit (Roche, USA, 91963). Monolayers were fixed in 4% paraformaldehyde for 30 min at room temperature, permeabilized with 0.5% Triton X-100 for 30 min, washed with PBST, and stained using the TUNEL Detection Kit (Beyotime, China, C1090). Images were acquired by fluorescence microscopy.

### Trans-epithelial electrical resistance measurement

Differentiated, mature human airway organoids grown on Transwell inserts under air–liquid interface (ALI) conditions were kindly provided by Dr. Guanghui Jin (The First Affiliated Hospital of Guangzhou Medical University, Guangzhou, China). siRNA transfection followed the A549 protocol. At 24 h post-transfection, trans-epithelial electrical resistance (TEER) was measured using a Millicell ERS2 (Millipore, USA) equipped with chopstick electrodes, according to the manufacturer’s instructions (22, 23). The measured resistance (Ω) was corrected by subtracting the blank insert and converted to TEER (Ω cm²) (24):


$$TEER=(measured\;resistance-blank\;resistance)\times membrane\;area\;(cm^{2}).$$

### Statistical analysis

Statistical analyses were performed in Prism (v9.0.0). Comparisons between two groups were performed using the unpaired two-tailed Student’s *t*-test. For analyses involving multiple groups, one-way ANOVA followed by Tukey’s *post hoc* test was utilized to assess significant differences. Survival curves were compared using the log-rank (Mantel–Cox) test, and *P* < 0.05 was considered statistically significant.

## RESULTS

### Influenza virus infection upregulates CTSS levels in the mouse lung

To determine the effects of influenza infection on the dynamic host responses in mouse lungs, 6–8-week-old female C57BL/6J mice were intranasally infected with serial doses (10–10,000 PFU) of A/Puerto Rico/8/1934 (H1N1) (PR8) or PBS. A dose-dependent increase in body weight loss was observed, with higher doses resulting in more significant weight loss (Fig. S1A). Survival rates were dose dependent: 83.3% (5/6) at 10 PFU, 16.7% (1/6) at 100 PFU, and 0% at 1,000 or 10,000 PFU (Fig. S1B). Consistently, viral loads in lungs reached a peak at 3 dpi, with higher viral loads correlating with increased infection doses (Fig. S1C). Hematoxylin and eosin (H&E) staining revealed progressive lung lesions and immune cell infiltration with increasing doses and over time from 1 to 7 dpi, with slight recovery noted at 14 dpi (Fig. S1D).

**Fig 1:**
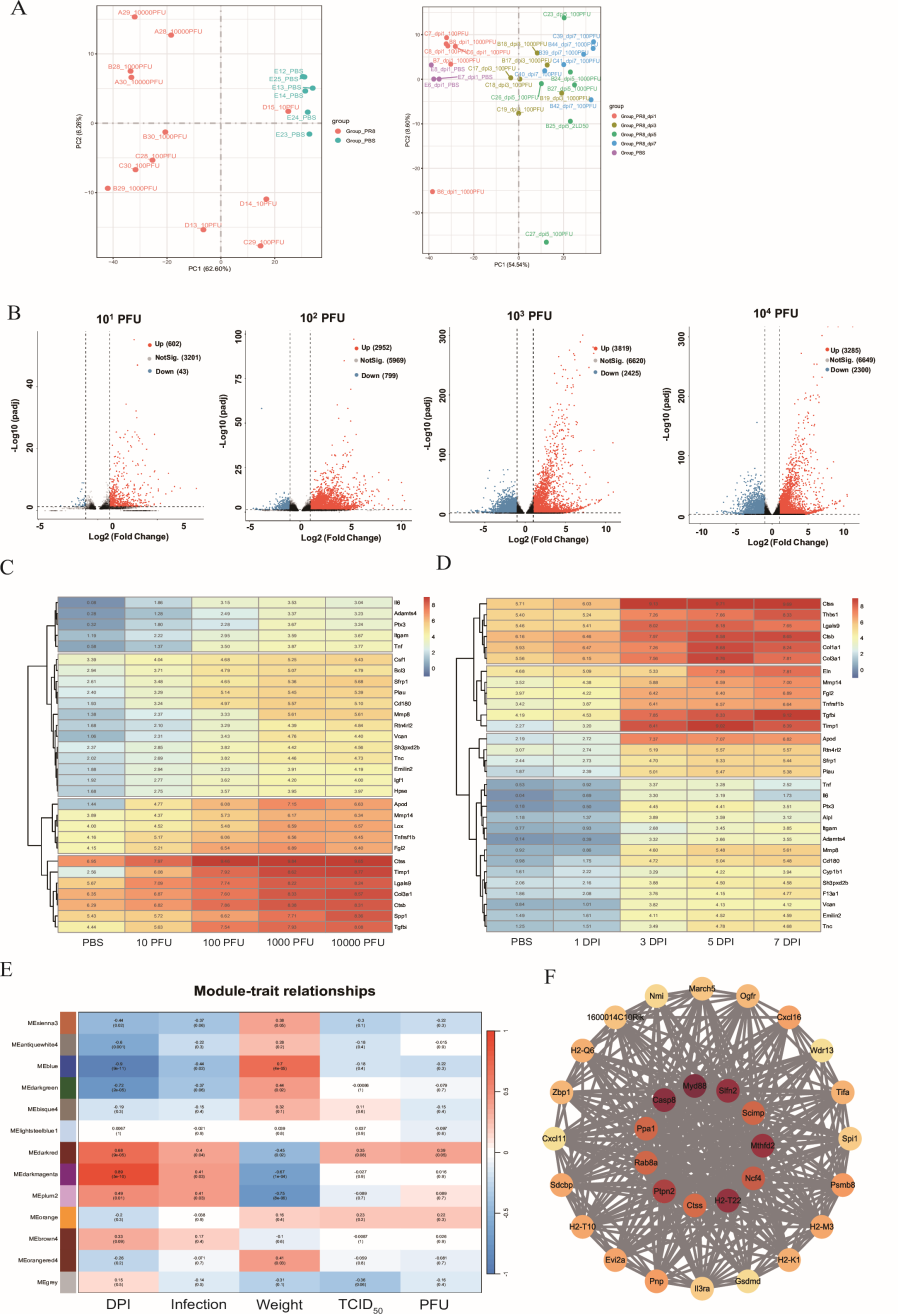
Pathogenesis dynamics and transcriptomic changes during influenza infection in mice. (**A**) Left: the PCA for the sequencing samples at 5 dpi with various infection dose. Right: the PCA for the sequencing samples with 100 or 1,000 PFU PR8 IAVs infection during the time post-infection (1, 3, 5, and 7 dpi). (**B**) Volcano plots of the DEGs between PBS and IAVs treated groups (10^1^, 10^2^, 10^3^, and 10^4^ PFU, respectively). The abscissa represents the log_2_FC, the ordinate represents the −log_10_ (*P* adj). Red points are the upregulated DEGs defined by the absolute value of FC > 2 and *P* adj < 0.05, while blue points are the downregulated DEGs. (**C and D**) Heatmap clustering of key extracellular-matrix-related genes from overlap DEGs across various infection doses at 5 dpi (**C**) and across multiple timepoints in the 100 PFU infection group (**D**). Expression levels are shown as log_2_-transformed FPKM values after centralization correction, highlighting the top 30 genes with significant changes relative to the PBS group. (**E**) WGCNA module identification and trait correlation, positive and negative correlations are indicated by red and blue colors, respectively. (**F**) Gene interaction network within the ME-darkred module, where node color depth indicates the number of connections. The central area highlights candidate hub genes.

To identify key host factors in acute lung injury caused by influenza A virus (IAV) infection, we performed lung transcriptomics across doses and time points. Principal component analysis (PCA) is shown in Fig. 1A. DEGs (log_₂_-centered FPKM) versus PBS controls are summarized in volcano plots (Fig. 1B). As ECM-associated protease activity is important for lung function after severe respiratory infection, we evaluated ECM-related gene regulation; heat maps of the top 30 ECM-related genes across dose and time are shown in Fig. 1C and D. Among these ECM-related proteases, CTSS—a lysosomal cysteine proteinase—displayed the most prominent upregulation, correlating with dose and duration of infection. In order to further validate the key function of DEGs involved in lung injury caused by influenza infection in mice, DEGs were grouped into 13 modules using WGCNA (Fig. 1E). Of note, ME-darkred was positively correlated with infection status, time post-infection, and viral titer, and negatively correlated with weight change, indicating a potential role in disease progression. GO enrichment analysis and protein interaction network analyses showed that this module was enriched for antiviral immune responses and apoptosis-related pathways (Fig. S2). Hub genes included CTSS, Myd88, Caspase8, Sifn2, and Mthfd2 (Fig. 1F).

**Fig 2:**
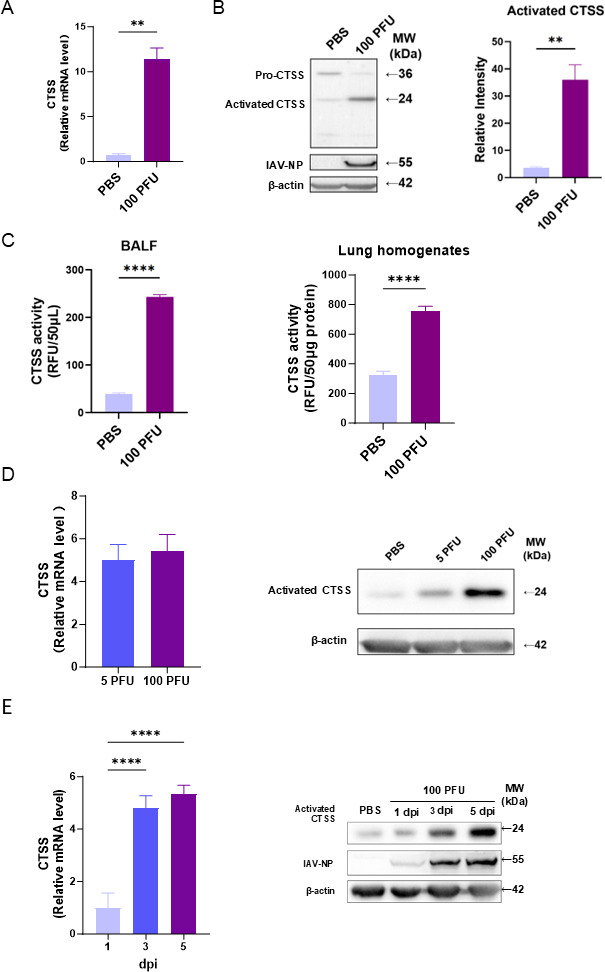
CTSS is upregulated by influenza infection in mouse lung. (**A**) CTSS mRNA expression in lung tissues from mice (*n* = 3 per group) inoculated with either PBS or 100 PFU of PR8 virus, quantified by RT-qPCR. (**B**) Western blotting analysis of proteins in lung tissues from mice inoculated with PBS or 100 PFU of PR8 virus. (**C**) CTSS activity in BALF and lung homogenates from mice inoculated with PBS or 100 PFU of PR8, measured using a CTSS Activity Assay Kit. (**D**) Left panel: comparison of CTSS mRNA expression levels in the lungs of mice infected with 5 PFU and 100 PFU of IAV, determined by RT-qPCR. Right panel: western blotting analysis of activated CTSS protein levels in lung tissues from mice infected with 5 PFU and 100 PFU of PR8. (**E**) Temporal profile of CTSS mRNA expression across various days post-infection, measured via RT-qPCR (left), and western blotting analysis of activated CTSS protein levels across different timepoints post-infection (right). Data are represented as mean ± standard deviation (SD). **, *P* < 0.01; ***, *P* < 0.001; ****, *P* < 0.0001.

We validated CTSS upregulation and activation in the lungs of mice infected with 100 PFU PR8. RT-qPCR showed significantly increased CTSS mRNA (Fig. 2A). Western blotting showed decreased pro-CTSS and increased activated CTSS (Fig. 2B). Moreover, CTSS activity in both BALF and lung tissue homogenates was significantly higher than in PBS controls (Fig. 2C). Activated CTSS further increased with dose and time (Fig. 2D and E), consistent with transcriptomic patterns.

### CTSS inhibition alleviates influenza virus-induced lung injury in mice

**Fig 3:**
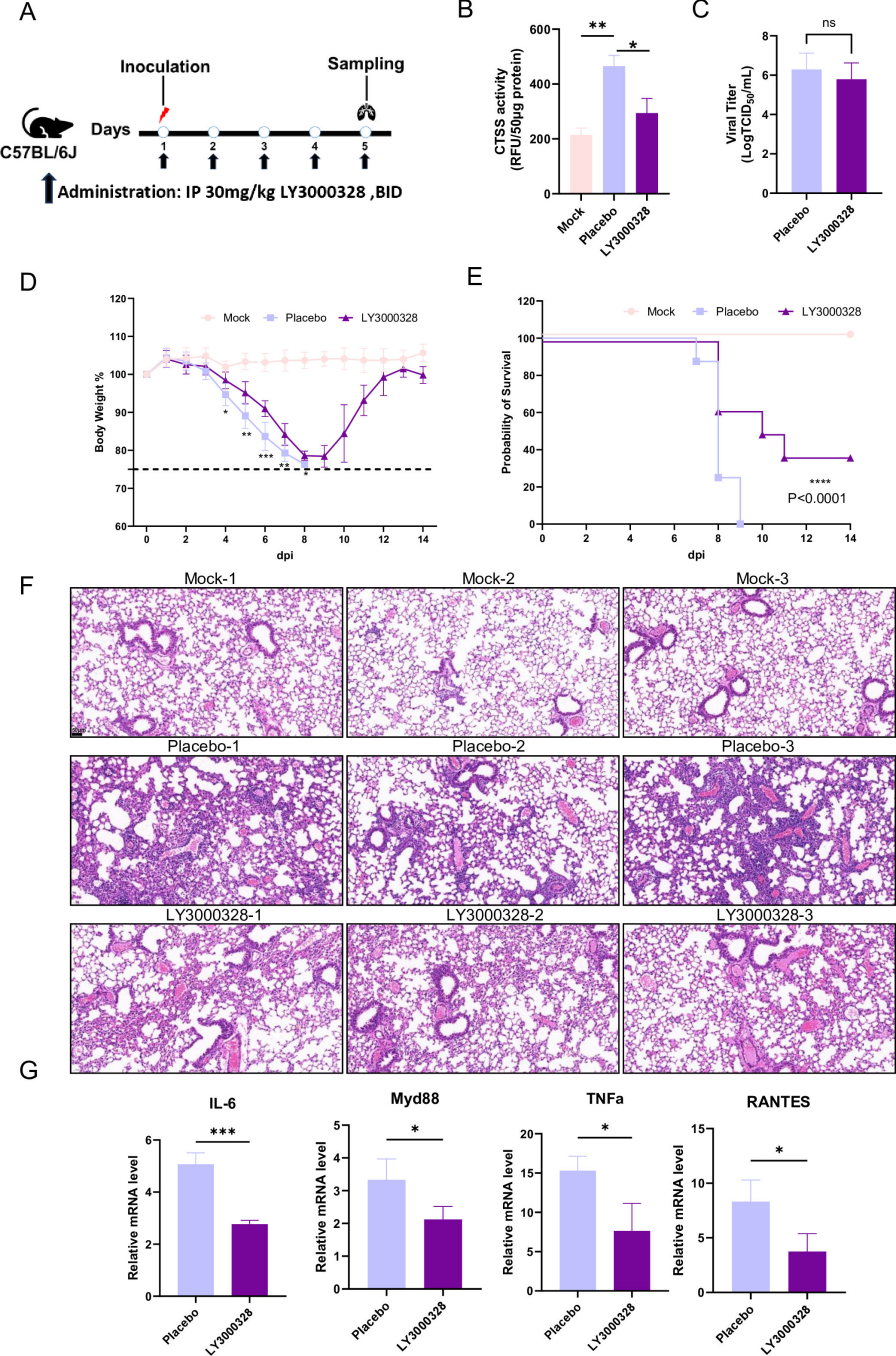
The effects of CTSS inhibitor LY3000328 on influenza infection in mice. (**A**) Schematic diagram of the experimental design. C57BL/6J mice (*n* = 5 per group) received intraperitoneal injection of 30 mg/kg LY3000328 or a placebo vehicle twice daily for 5 days. Four hours after the initial dose, mice were intranasally inoculated with 100 PFU of PR8 virus. A mock control group received the vehicle orally twice daily and PBS intranasally. Body weight was monitored daily. Mouse lungs were collected at 5 dpi for the analyses of viral titers, histopathology, and gene expression. (**B**) CTSS activity in lung homogenate among different groups. (**C**) Viral titers in lung homogenates determined using the TCID_50_ assay in MDCK cells. (**D**) Body weight change of mice across the experimental period. (**E**) Survival curve of mice across the experimental period. (**F**) Histopathological examination of mouse lungs performed by H&E staining. (**G**) Analysis of mRNA expression levels of cytokines in lung tissues by RT-qPCR. Data are represented as mean ± SD. *, *P* < 0.05; **, *P* < 0.01; ***, *P* < 0.001; ns, non-significant.

To evaluate whether CTSS inhibition attenuates influenza virus-induced pulmonary injury, mice were infected with 100 PFU PR8 and treated with the CTSS inhibitor LY3000328 (30  mg/kg, intraperitoneally, twice daily) or vehicle, mock mice received the vehicle and PBS (Fig. 3A). CTSS activity in lung tissues was assessed at 5 dpi. Compared with the mock group, CTSS activity was significantly elevated in the placebo group following infection, whereas LY3000328 treatment significantly suppressed CTSS activity relative to the placebo group (Fig. 3B). Despite similar lung viral loads at 5  dpi across both infected groups (Fig. 3C), notable differences in disease progression were observed. Placebo-treated mice exhibited progressive body weight loss from 3  dpi and began dying at 7  dpi, reaching 100% mortality by 9  dpi. While LY3000328-treated mice showed milder body weight loss, with partial weight recovery by 10 dpi, and 37.5% mice survived to 14  dpi (Fig. 3D and E).

The lung lesions in the LY300328-treated mice were obviously milder than in the placebo group (Fig. 3F), consistent with the differences in body weight loss between the two groups. Additionally, the mRNA expression level of pro-inflammatory cytokines and chemokines (i.e., Myd88, IL-6, TNF-α, CCL-5/RANTES) in lung tissues collected at 5 dpi was determined (Fig. 3G). Results showed that, compared to the placebo group, influenza-infected mice treated with LY3000328 had significantly lower mRNA expressions of cytokine and chemokine in the lungs.

### CTSS does not affect PR8 virus replication but reduces cytokine expression in A549 cells

**Fig 4:**
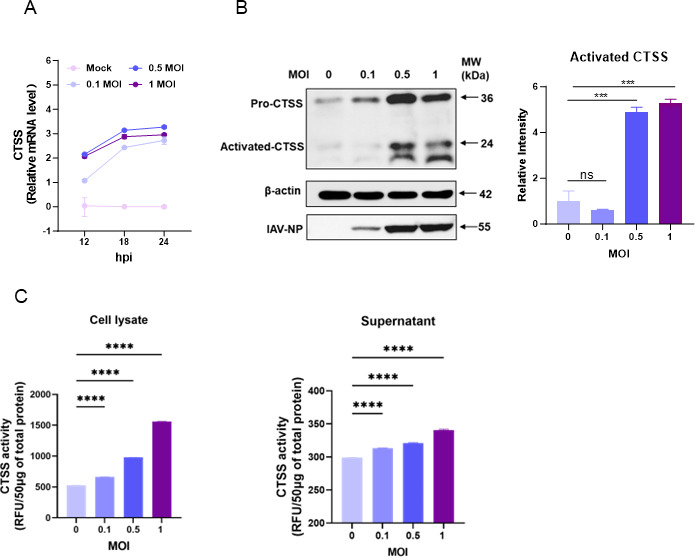
Influenza infection activates CTSS expression in lung epithelial cells (A549). (**A**) CTSS mRNA expression in A549 cells following infection with PR8 virus at MOIs of 0, 0.1, 0.5, and 1. (**B**) Protein levels of pro-CTSS and activated CTSS in A549 cells 24 hpi with serial MOIs of PR8 virus, assessed by western blotting. (**C**) CTSS activity in the supernatant and cell lysate of A549 cells infected with indicated MOI of PR8 virus at 24 hpi, measured using a CTSS Activity Assay Kit. Data are presented as mean ± SD from three independent experiments. ***, *P* < 0.001; ****, *P* < 0.0001; ns, non-significant.

To determine the role of CTSS in influenza infection-induced lung lesions, we first determined the expression and activity of CTSS in influenza virus-infected human lung epithelial (A549) cells. CTSS mRNA levels in A549 cells infected with different MOI of influenza virus were assessed at 12, 18, and 24 hpi (Fig. 4A). The results showed a significant increase in CTSS mRNA expression in a dose-dependent and time-dependent manner, with higher MOIs (0.5 and 1) showing greater increases in CTSS expression compared to mock and lower MOIs (0.1). Similarly, compared to the mock control, both pro-CTSS and activated CTSS protein levels were elevated in the influenza-infected A549 cells, with a trend of increasing CTSS protein levels corresponding to higher MOIs, which aligned with higher viral NP protein levels (Fig. 4B). Furthermore, CTSS enzymatic activity in A549 cell lysates increased in a dose-dependent manner after infection, and the activity of CTSS in the cell supernatant was also slightly increased (Fig. 4C).

**Fig 5:**
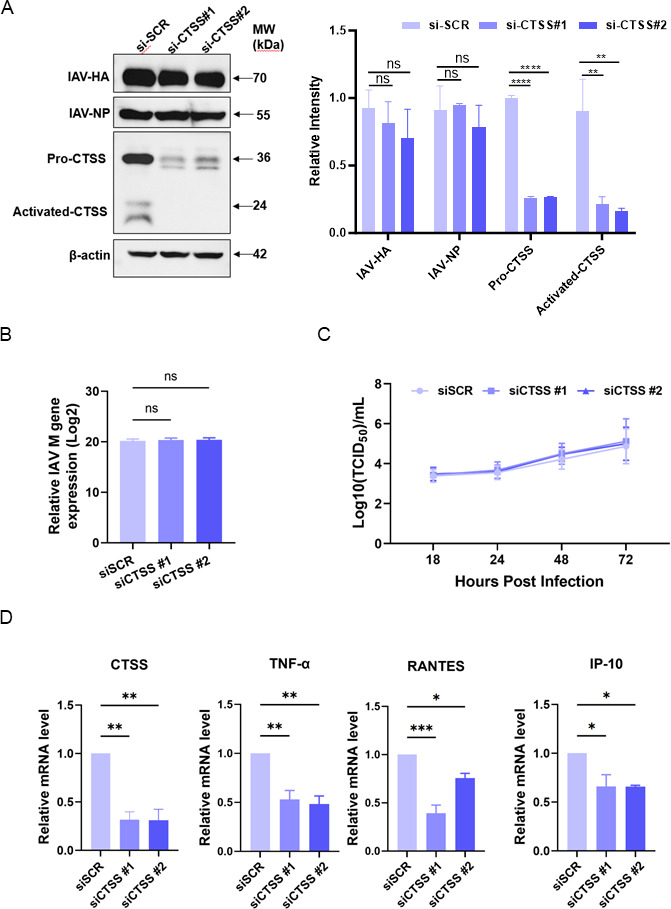
CTSS does not affect PR8 virus replication but reduces cytokine expression in A549 cells. A549 cells were transfected with two different CTSS-siRNAs (siCTSS #1 and siCTSS #2) or a scramble siRNA (siSCR) and then infected with 0.25 MOI of PR8 virus. (**A**) Left panel: representative western Blot analysis of CTSS and viral proteins (HA and NP) expression at 24 hpi, Right panel: intensity scanning of the bands from the left, quantified with data from three independent repeats. (**B**) Viral M gene expression at 24 hpi was quantified by RT-qPCR. (**C**) Viral titers at specified timepoints were measured using the TCID_50_ assay. (**D**) The changes in mRNA expression levels of CTSS, TNF-α, RANTES, and IP-10 at 24 hpi were assessed by RT-qPCR. Data are presented as mean ± SD. Two to three independent experimental repeats were conducted for each assay. *, *P* < 0.05; **, *P* < 0.01; ***, *P* < 0.001; ****, *P* < 0.0001; ns, non-significant.

Treatment of A549 cells with different concentrations of recombinant human CTSS protein during influenza virus infection significantly enhanced the mRNA expression of virus-induced inflammatory genes (Fig. S3A). To test effects on viral replication, CTSS was knocked down by siRNAs (siCTSS #1 and #2), both of which reduced CTSS protein (Fig. 5A). However, neither viral mRNA (M gene), viral proteins (NP and HA), nor viral titers were altered by CTSS knockdown (Fig. 5A through C). In contrast, the mRNA expression of TNF-α, RANTES, and IP-10 was downregulated after CTSS knockdown, consistent with the CTSS inhibition experiment *in vivo* (Fig. 5D).

### CTSS is released into the cytoplasm during influenza infection and promotes apoptosis

**Fig 6:**
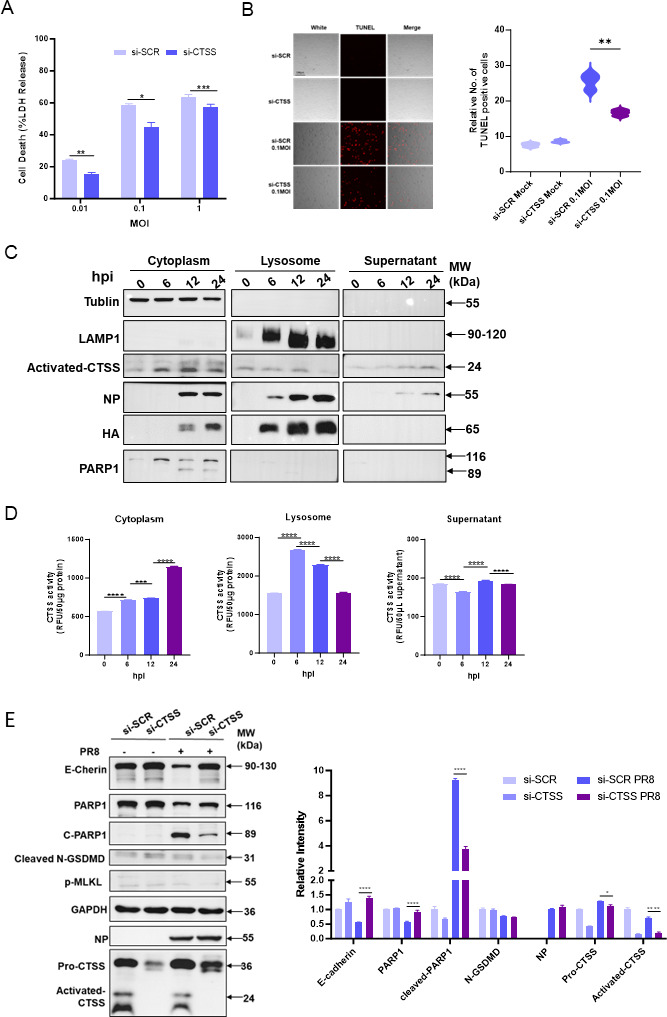
CTSS is released into the cytoplasm during influenza infection, thereby regulating apoptosis and epithelial barrier integrity. (**A**) Cell death was assessed by measuring LDH release from non-targeting and CTSS-targeting siRNA transfected A549 cells following PR8 infection at 24 hp i, using an LDH Cytotoxicity Assay Kit (Roche, USA, 91963) according to the manufacturer’s instructions. (**B**) TUNEL staining and quantification of positive cells in non-targeting and CTSS-targeting siRNA-transfected A549 cells after PR8 virus infection at 24 hpi. Representative images (left panel) and statistical data (right panel) are shown. (**C**) Protein levels of CTSS, LAMP1, NP, and HA and PARP1 in the cytoplasm, lysosome, and supernatant of A549 cells infected with PR8 (MOI = 0.1) at 0, 6, 12, and 24 hpi were determined by western blotting. (**D**) CTSS activity in the cytoplasm, lysosome, and supernatant of A549 cells infected with PR8 virus was measured using a CTSS Activity Assay Kit. (**E**) Expression of CTSS, intercellular tight junctions, and cell death markers in non-targeting and CTSS-targeting siRNA transfected A549 cells following PR8 virus infection at 24 hpi was determined by western blotting. Data are represented as mean ± SD. *, *P* < 0.05; **, *P* < 0.01; ***, *P* < 0.001; ****, *P* < 0.0001.

Given the observed correlation between programmed cell death and CTSS upregulation from WGCNA (Fig. 1F), we evaluated the cell death in A549 cells after CTSS knockdown. Our findings revealed that CTSS knockdown significantly reduced cell death caused by influenza virus infection through detecting lactate dehydrogenase (LDH) released in the cell supernatant (Fig. 6A). Similarly, TUNEL assay confirmed that CTSS knockdown significantly reduced TUNEL-positive cells caused by influenza infection (Fig. 6B).

Because influenza virus infection can induce lysosomal membrane permeabilization (LMP) (25), lysosomal contents may be released into the cytoplasm, causing cell stress and damage. To determine whether CTSS—a lysosome-resident cathepsin markedly upregulated after influenza infection—undergoes subcellular redistribution, we examined subcellular CTSS distribution and activity over time. At the 6 hpi, lysosomal CTSS activity was significantly elevated, whereas cytoplasmic activity remained low. From 12–24 hpi, lysosomal CTSS expression and activity declined, whereas cytoplasmic CTSS protein and activity increased, coinciding with elevated cleaved-PARP1, indicating the induction of apoptosis. Extracellular CTSS also rose modestly. These data suggest that activated CTSS is progressively released from lysosomes into the cytoplasm during infection, where it contributes to apoptosis (Fig. 6C and D).

To identify the predominant form of cell death, A549 cells were infected with increasing MOIs and analyzed for apoptosis (cleaved-PARP1), necroptosis (p-MLKL), and pyroptosis (cleaved-GSDMD). Cleaved-PARP1 increased dose-dependently, while p-MLKL and cleaved-GSDMD showed no consistent trends, indicating that apoptosis was the major pathway triggered by influenza infection in A549 cells (Fig. S4). Accordingly, siRNA-mediated CTSS knockdown significantly reduced PARP1 cleavage without altering MLKL phosphorylation or GSDMD cleavage in A549 cells (Fig. 6E). Moreover, treatment with recombinant CTSS protein enhanced influenza-induced PARP1 cleavage in a dose-dependent manner (Fig. S3B), further supporting a pro-apoptotic role for CTSS during infection.

### CTSS disrupts epithelial barrier integrity in human airway organoids

Because CTSS activity increased in supernatants after infection (Fig. 4C) and CTSS targets ECM and junctional components, we tested effects on epithelial barrier function. Western blotting analysis in A549 cells revealed that influenza virus infection led to a dose-dependent reduction in E-cadherin expression (Fig. S4), indicating disruption of cell–cell adhesion and compromise of barrier integrity. It is worth noting that siRNA-mediated knockdown of CTSS significantly increased the expression level of E-cadherin and reversed the decrease in E-cadherin levels induced by influenza virus (Fig. 6E).

**Fig 7:**
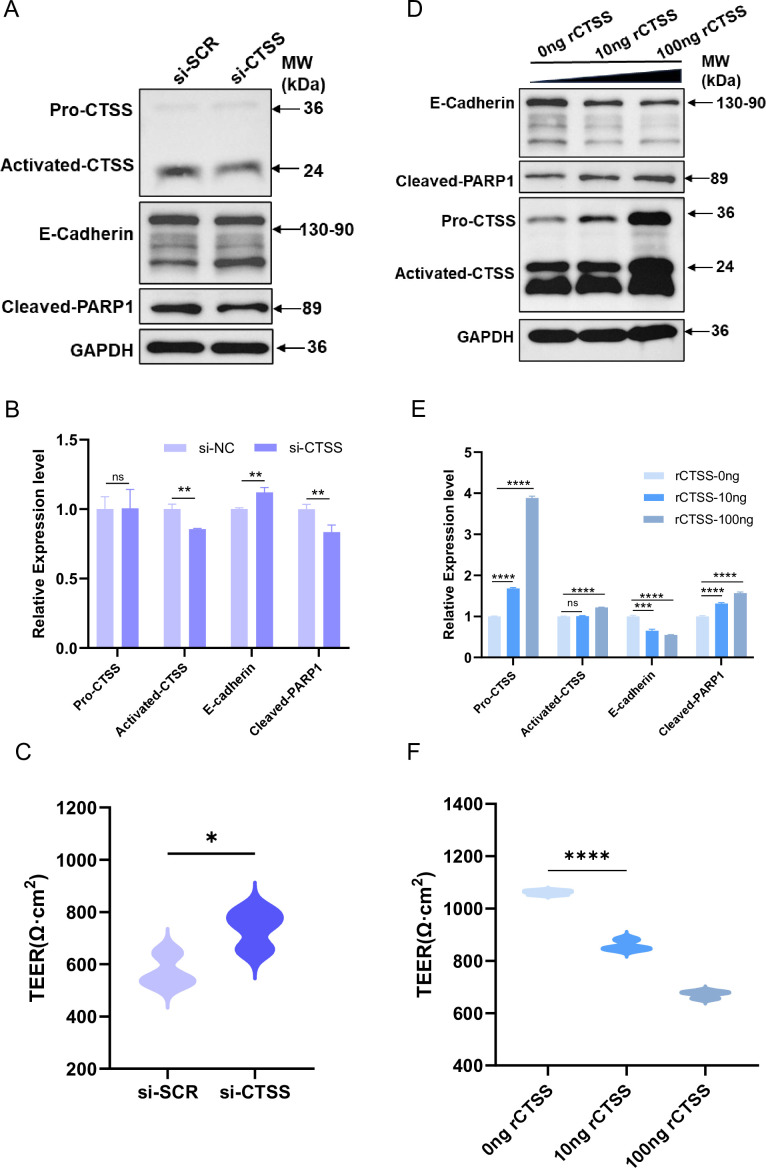
Role of CTSS in maintaining the epithelial barrier integrity of human airway organoids. (**A, B**) Western blotting was performed to detect protein levels in non-targeting and CTSS-targeting siRNA transfected human airway organoids. (**A**) Representative images and statistical data (**B**) are shown. (**C**) TEER value measured in non-targeting and CTSS-targeting siRNA transfected human airway organoids. (**D, E**) Western blotting was performed to detect protein levels in rCTSS protein treated human airway organoids. (**D**) Representative images and statistical data (**E**) are shown. (**F**) TEER value measured in rCTSS protein treated human airway organoids. Data are represented as mean ± SD. *, *P* < 0.05; **, *P* < 0.01; ***, *P* < 0.001; ****, *P* < 0.0001.

To further assess the functional impact of CTSS on epithelial barrier properties, differentiated airway organoids were transfected with CTSS-targeting siRNA. Twenty-four hours after transfection, TEER was measured, and organoids were lysed for western blotting analysis. Compared with the non-targeting control, CTSS knockdown produced a modest but significant increase in TEER (Fig. 7C). Consistently, western blotting confirmed reduced levels of activated-CTSS and cleaved-PARP1, accompanied by a slight enhancement of E-cadherin expression in the airway organoids (Fig. 7A and B).

Because siRNA-mediated knockdown efficiency in organoids was limited, recombinant CTSS (rCTSS) protein was added to differentiated airway organoids to further evaluate the role of CTSS in epithelial barrier function. Exogenous rCTSS at 10 ng and 100 ng caused a dose-dependent decrease in TEER (Fig. 7F) and significantly increased activated-CTSS and cleaved-PARP1, while reducing E-cadherin levels (Fig. 7D and E). These results demonstrate that CTSS compromises tight-junction integrity and epithelial barrier function, and they provide functional confirmation of the siRNA data.

Collectively, these data indicate that lowering CTSS activity helps preserve epithelial barrier integrity and intercellular junctions during influenza infection.

## DISCUSSION

In this study, we demonstrated that CTSS is markedly upregulated and activated during influenza virus infection and plays a critical role in modulating host inflammatory responses and epithelial barrier integrity rather than directly affecting viral replication. In addition, we found that high-dose infection further increased the accumulation of activated-CTSS (Fig. 2E and 3A). We propose that stronger viral replication and inflammation at higher doses enhance lysosomal activity, driving the conversion of pro-CTSS to its active form. Moreover, elevated proinflammatory cytokines such as TNF-α and interferon (IFN)-γ may additionally promote CTSS processing and activation, changes that are less likely to occur during low-dose infection.

Activity inhibition of CTSS in mice did not alter pulmonary viral titers but significantly alleviated lung inflammation, reduced disease severity, and improved survival. Similarly, siRNA-mediated knockdown of CTSS in A549 cells led to decreased production of proinflammatory cytokines, reduced cleavage of the apoptotic marker PARP1, and preserved E-cadherin expression, thereby maintaining tight junctions and epithelial barrier function during infection. Conversely, treatment with recombinant CTSS protein exacerbated IAV-induced PARP1 cleavage and cytokine expression in A549 cells, indicating that CTSS acts as an amplifier of influenza-induced inflammatory and pathological injury process.

ECM-associated proteases are increasingly recognized as key regulators of host responses and viral pathogenesis (26). The ECM comprises structural proteins and several proteases that remodel these proteins (27). We previously reported that the ECM protease ADAMTS4 mediates lung injury during influenza infection (10). Here, transcription analysis highlighted CTSS among eight major human cathepsins: only cathepsin W (CTSW) and CTSS were significantly upregulated, whereas others were downregulated or unchanged (Fig. S5), suggesting unique functions. CTSW is known to facilitate influenza virus release from late endosome (28), although CTSS is an interferon-stimulated gene upregulated by IFN-γ (29), its role in influenza-induced tissue injury had not been elucidated.

Cathepsins can affect viral infection either directly—by targeting viral proteins and altering the infection cycle—or indirectly—by modulating host immune responses. For example, CTSB and CTSL cleave viral glycoproteins to facilitate entry of Ebola virus (30, 31); CTSB participates in human papillomavirus type 16 entry (32); CTSL and CTSS support reovirus infection in an acid-independent manner (33, 34); and CTSL mediates spike protein cleavage in coronaviruses, including SARS-CoV, MERS-CoV, and SARS-CoV-2 (35, 36). Therefore, we explored the mechanism of CTSS in influenza infection.

Mechanistically, we observed subcellular redistribution of CTSS from lysosomes into the cytoplasm during the late stages of infection, consistent with LMP (37). Lysosomal leakage is a well-established trigger of apoptosis and has been reported for dengue virus, Newcastle disease virus, and coronaviruses including SARS-CoV, MERS-CoV, and SARS-CoV-2 (38–40). Our data suggest that accumulated CTSS in the cytoplasm contributes to apoptotic activation, leading to extensive epithelial cell death and barrier disruption. Knockdown of CTSS significantly reduced inflammation and apoptosis, preserved epithelial barrier function, and mitigated lung injury. Importantly, TNF-α robustly induced CTSS expression and activation; however, in CTSS-knockdown cells, TNF-α failed to restore apoptosis or inflammatory responses (Fig. S6), indicating that CTSS is a critical downstream effector of TNF-α-driven immunopathology.

The role of CTSS in epithelial barrier disruption was further validated in air–liquid interface (ALI) differentiated human airway organoids, which recapitulate airway architecture and barrier properties and have been widely used to study viral infection and epithelial dysfunction (41, 42). CTSS knockdown significantly increased TEER and restored E-cadherin expression, indicating improved tight junction integrity and epithelial cohesion during infection. These findings provide strong experimental evidence that CTSS directly contributes to epithelial barrier breakdown in the context of viral infection.

Given the contribution of cytokine storm to severe influenza outcomes (43, 44), our findings position CTSS as a host-derived pathogenic mediator integrating viral and cytokine-driven injury pathways. Targeting CTSS may therefore offer a promising therapeutic strategy to mitigate immunopathology. Indeed, CTSS has been proposed as a biomarker and therapeutic target in diverse pulmonary diseases (45), and preclinical studies in cystic fibrosis and COPD models show that CTSS inhibition alleviates mucus hypersecretion, inflammation, and functional decline (46–48). These observations support the translational potential of CTSS-targeted interventions for influenza-induced lung injury.

Nevertheless, while *in vitro* models such as A549 cells and air–liquid interface (ALI) differentiated airway epithelial cultures provide valuable insight, they cannot fully recapitulate the complex lung microenvironment, including immune cell crosstalk and extracellular matrix interactions. Future investigations addressing these limitations will be critical to validate CTSS as a viable therapeutic target and to deepen our understanding of its role in respiratory viral pathogenesis.

In summary, our findings identify CTSS as a pivotal mediator linking influenza virus infection to excessive inflammation, apoptosis, and epithelial barrier dysfunction. Targeting CTSS holds promise as a host-directed therapeutic approach to improve clinical outcomes in severe influenza, warranting further investigation and development.

## Data Availability

Sequence data are available in the NCBI Sequence Read Archive under BioProject accession number PRJNA1274851.

## SUPPLEMENTAL MATERIAL

Supplemental material spectrum.01128-25-s0001.pdf Figures S1 to S6; Table S1.

ASM does not own the copyrights to Supplemental Material that may be linked to, or accessed through, an article. The authors have granted ASM a non-exclusive, world-wide license to publish the Supplemental Material files. Please contact the corresponding author directly for reuse.
