# Supplementary material for: Cathepsin S contributes to influenza-induced lung injury by driving inflammation, promoting apoptosis, and disrupting epithelial barrier integrity
Source: Microbiol Spectr. 2025 Nov 19;14(1):e01128-25. doi: 10.1128/spectrum.01128-25 (PMC12772400; doi:10.1128/spectrum.01128-25)
Supplement: Supplemental material — Figures S1 to S6; Table S1. [file spectrum.01128-25-s0001.pdf]

Supplementary Figure 1: Body weight curves (A) and survival rates (B) of C57B/6J mice (n=6 per group) following intranasal infection with indicated doses of A/Puerto Rico/8/1934 (H1N1) (PR8) virus or with equal volume PBS as negative. (C) Virus titers in lung homogenates were measured at designated timepoints (n=3 per group). (D) H&E s staining of lung tissues from control and various dose PR8 -infected mice at 5 dpi as well as lung tissues from 100 PFU PR8 infected mice collected at different time points. Magnification: ×50. Scale bars: 1000μm.

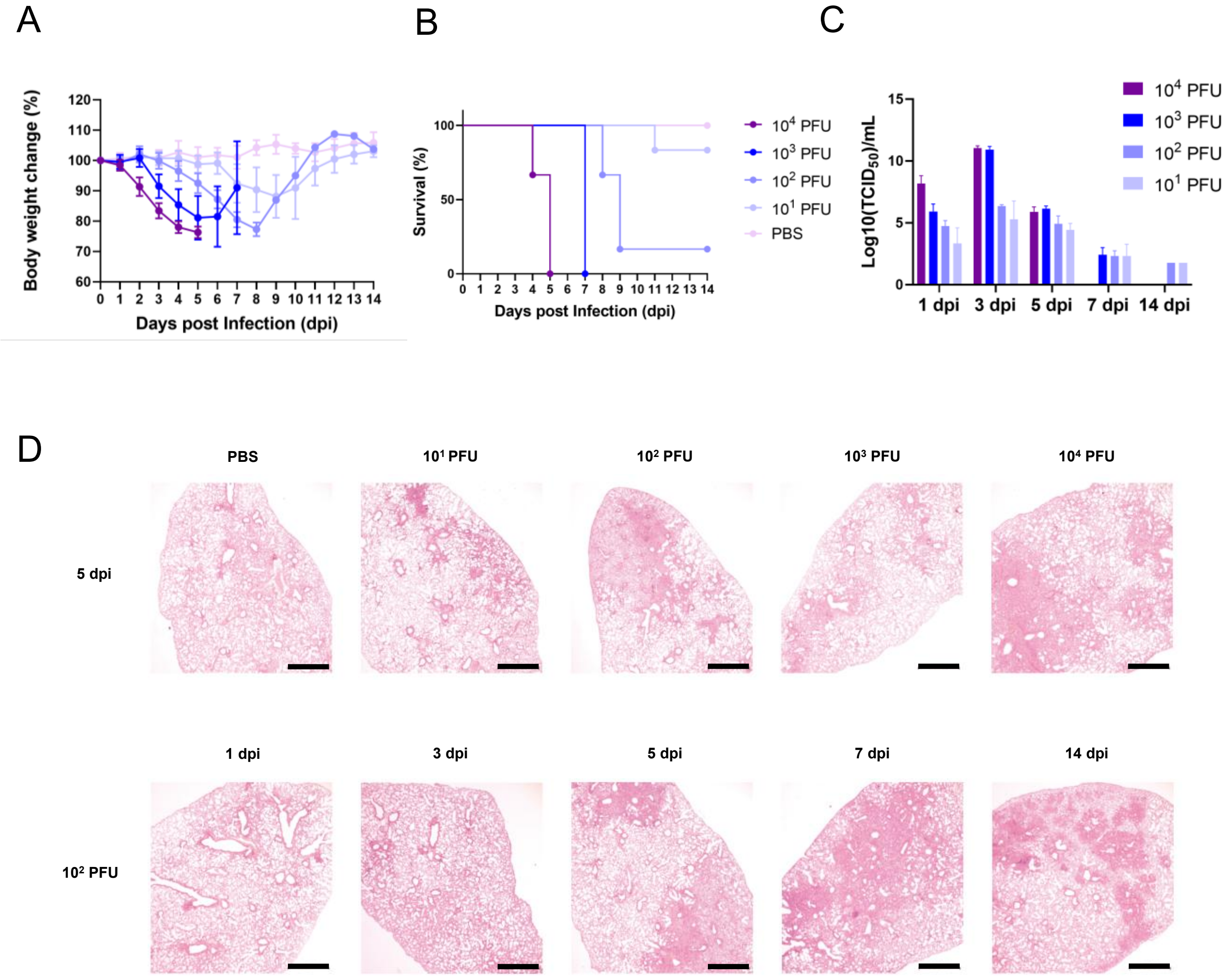

Supplementary Figure 2: (A) Graph of Power value. Left: the abscissa represents the power value, the ordinate represents the correlation coefficient, the red line represents the correlation coefficient of 0.8. Right: the abscissa represents the power value, and the ordinate represents the average connectivity of genes. (B) Heatmap of the network of initial 30 modules' Topological Overlap (TOM). Darker the color represented, the higher overlap among modules is. The gene tree diagram and module allocation are also displayed on the left and top of the heatmap. (C) Left panel: Biological process (BP) of gene GO annotation in the ME_darkred module. The graph demonstrates the top twenty terms by gene counts. Right panel: The significantly enriched pathways of the ME_darkred module from Kyoto Encyclopedia of Genes and Genomes (KEGG) database. The graph demonstrates the top twenty pathways by gene counts.

A

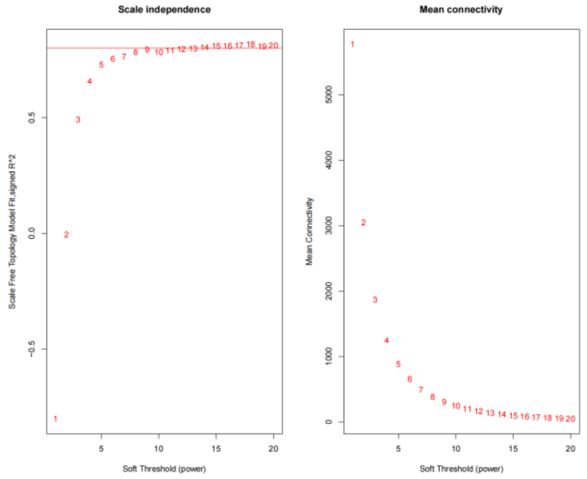

B

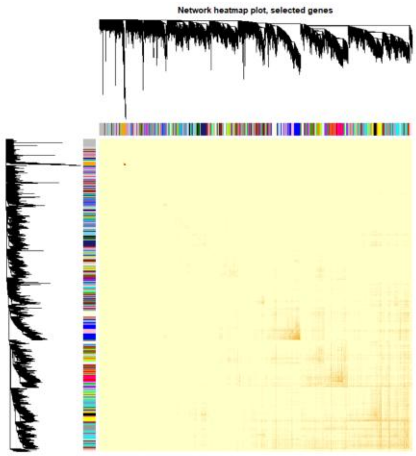

C

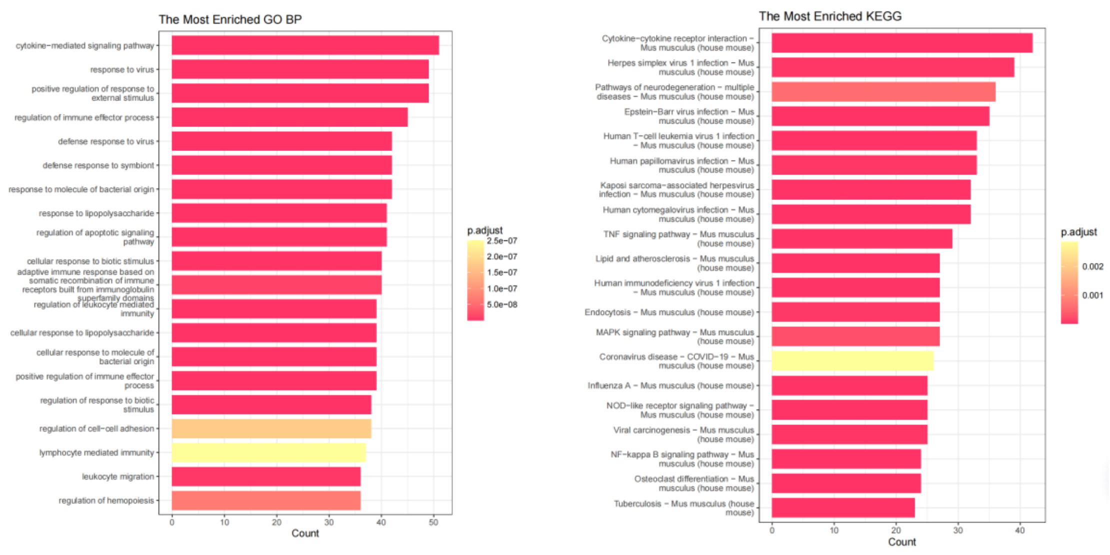

Supplementary Figure 3: A549 cells were infected with PR8 at 0.1MOI, and different doses of human-CTSS protein were added 1.5 hours post-infection. (A) Cells were harvested at 24 hpi and cytokines expression were detected by RT-qPCR. (B) Cells were lysed and proteins were collected at 24 hpi for detect the proteins related to cell death by western blotting.

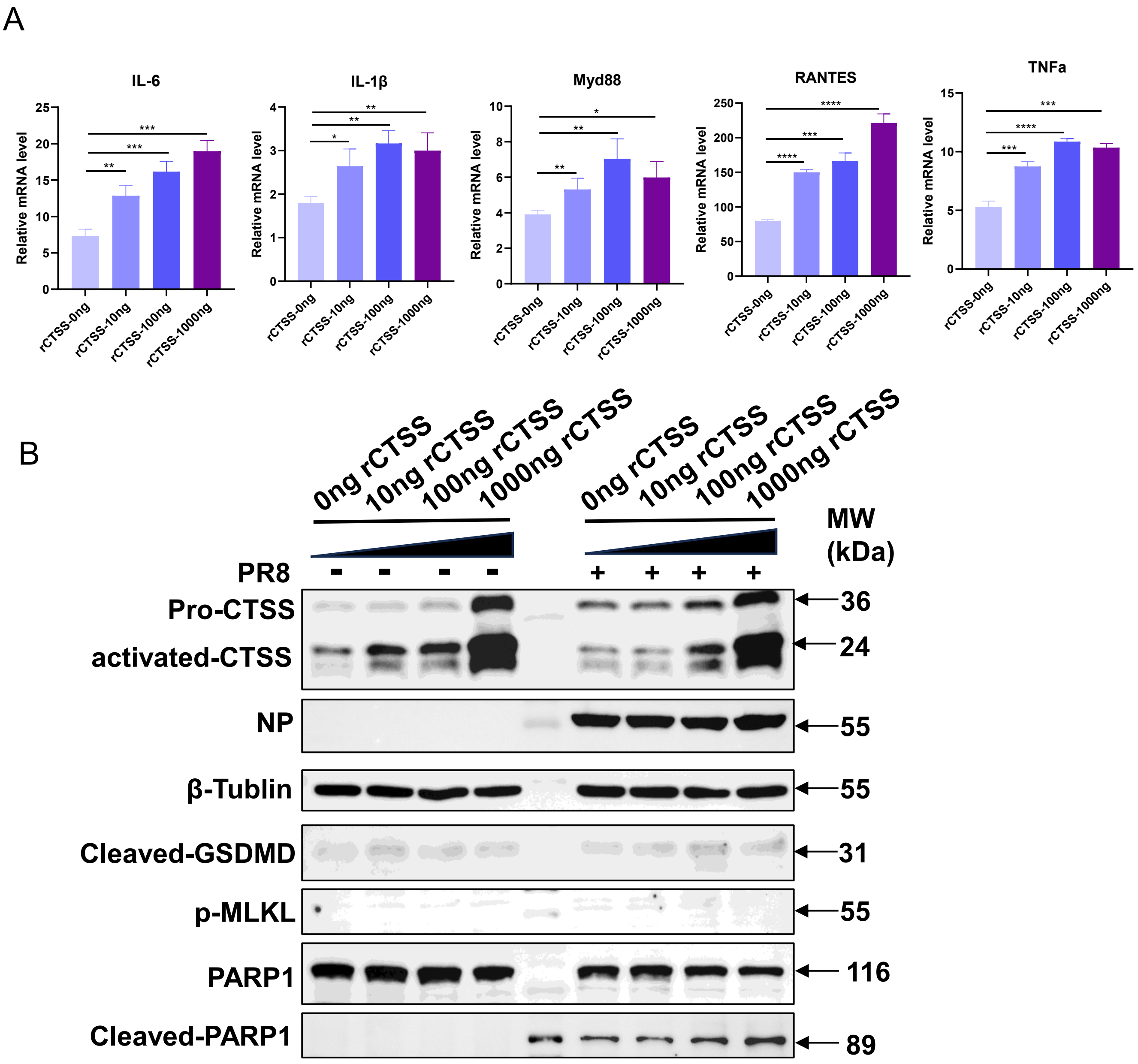

Supplementary Figure 4: A549 cells were infected with PR8 at different MOI, cell lysates were harvested at 24 hpi for detect the proteins related to cell death and intercellular junction by western blotting.

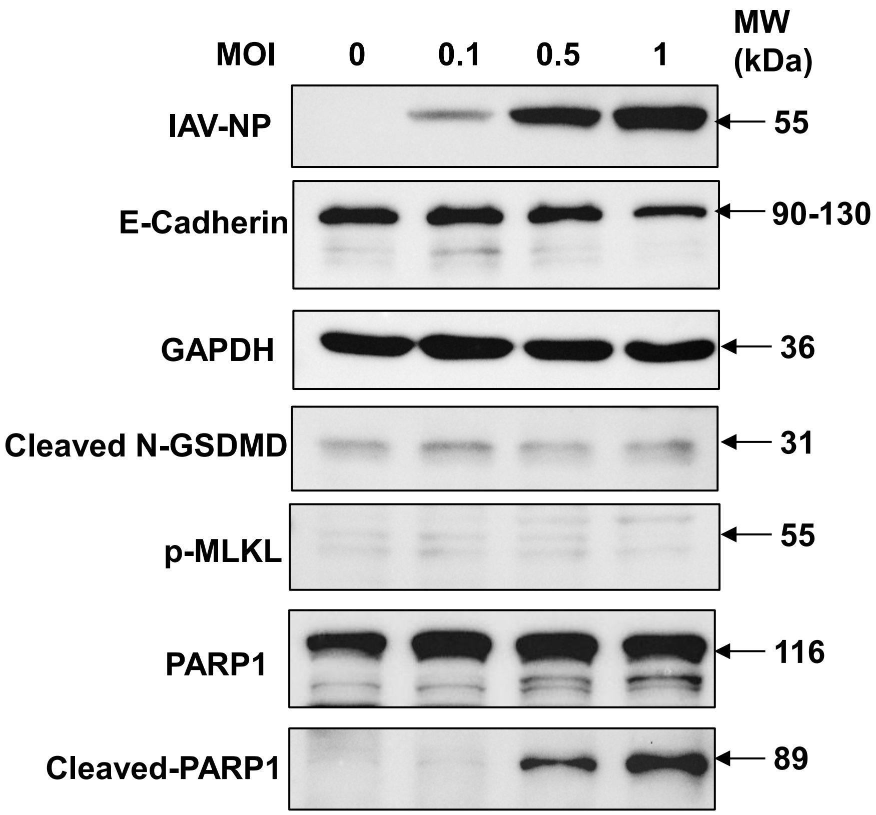

Supplementary Figure 5: A549 cells are infected with various dose of IAV. Gene expression of related cathepsins are detected by RT-qPCR.

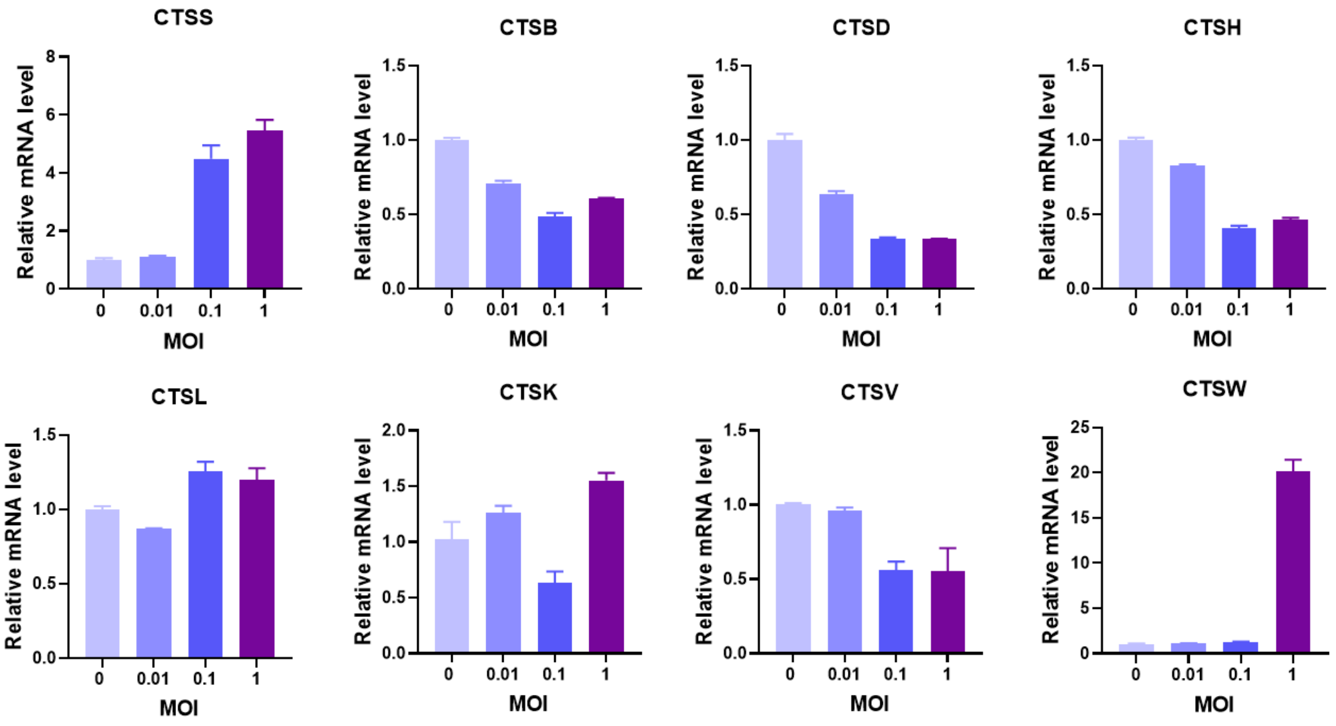

Supplementary Figure 6: A549 cells were respectively transfected with non-targeted or CTSS-targeted siRNA. After 24 hours transfection, cells were infected with PR8 at a MOI of 0.1. Then, 100 ng/mL human TNF-α protein was added 1.5 hours post infection. The cells were harvested and lysed 24 hours after infection. (A) Western blotting was used to detect the expression of death-related proteins. (B) qRT-PCR was used to detect inflammatory gene expression.

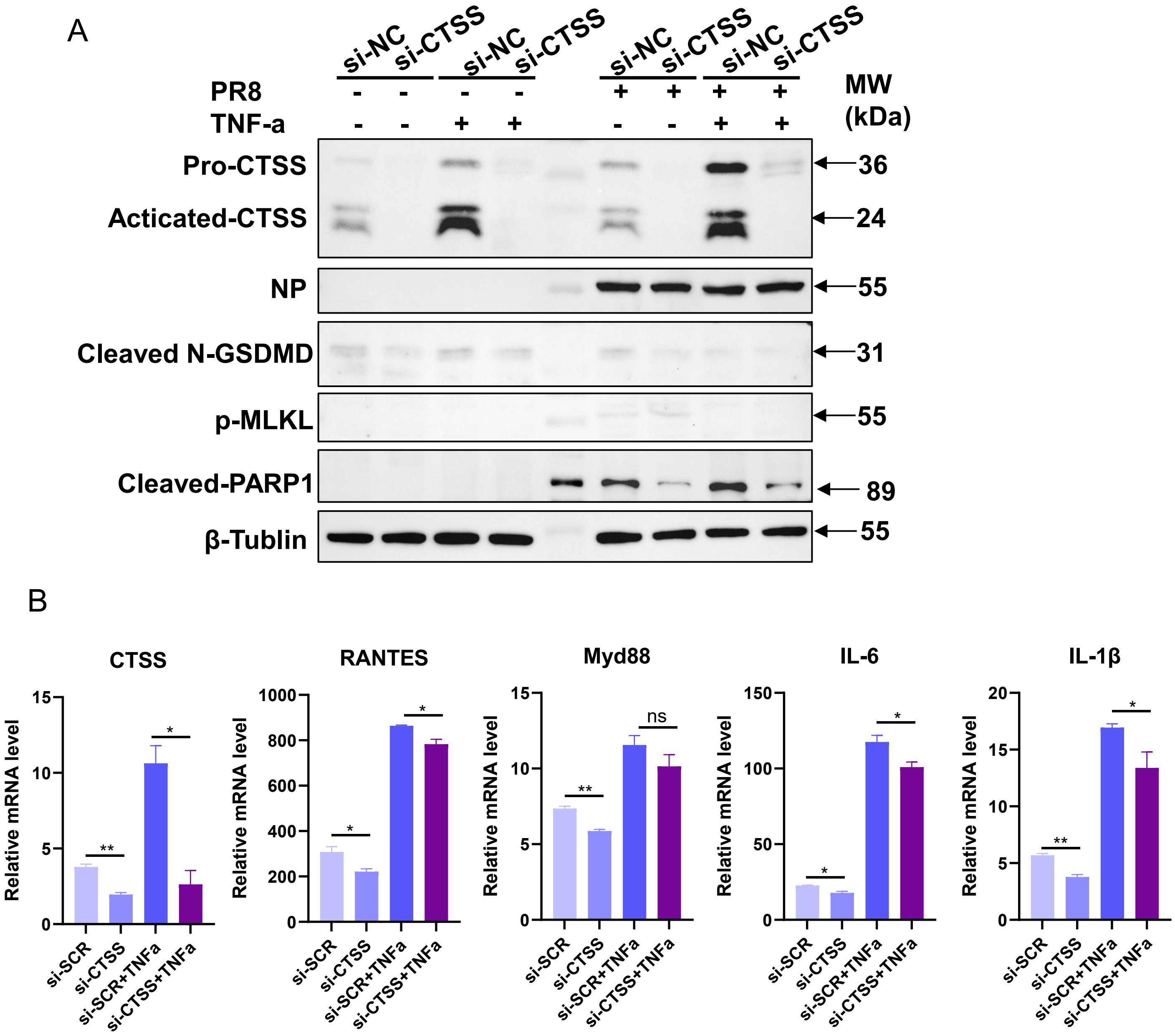

Supplementary Table 1

Primer sequences that were used for the Real-time qPCR

Gene	Forward primer (5'-3')	Reverse primer (5'-3')
IAV-M	ACGTTCTCTCTATCATCCCGTCA	CGTGAACACAAATCCTAAAATCCCCT
mCTSS	GAAGAAATCTTGTGTCGGATGG	CACAAGAACCCTGGTATTTCAC
mGAPDH	AACTTTGGCATTGTGGAAGG	GGATGCAGGGATGATGTTCT
mIL-6	AGTTGCCTTCTTGGGACTGA	GCCACTCCTTCTGTGACTCC
mRANTES	GCCCACGTCAAGGAGTATTT	CTTGAACCCACTTCTTCTCTGG
mTNFα	ACGTGGAACTGGCAGAAGAG	CTCCTCCACTTGGTGGTTTG
mMyd88	TCGATGCCTTTATCTGCTACTG	GGTCGGACACACACAACTTA
hGAPDH	CAAAATGGTGAAGGTCGGTGTG	GTTGAGGTCAATGAAGGGGTCG
hCTSS	ATGAAACGGCTGGTTTGTGTGC	GATTTCTGGGTAAGAGGGAAAGCTAGC
hRANTES	TGCCCACATCAAGGAGTATTT	GATGTACTCCCGAACCCATTT
hTNF-α	CCAGGGACCTCTCTCTAATCA	TCAGCTTGAGGGTTTGCTAC
hIP-10	TCCTGCAAGCCAATTTTGTCC	TCGATTTTGCTCCCCTCTGG
hIL-6	GGAGACTTGCCTGGTGAAA	CTGGCTTGTTCCTCACTACTC
hIL-1β	ATGGACAAGCTGAGGAAGATG	CCCATGTGTCGAAGAAGATAGG
hMyd88	CTGTGTCTGGTCTATTGCTAGTG	TTCCTTGCTCTGCAGGTAATC
hCTSB	GGACAAGCACTACGGATACAA	GTAGAGCAGGAAGTCCGAATAC
hCTSD	CGAGGTGCTCAAGAACTACAT	GTGTCGAAGACGACTGTGAA
hCTSH	CCAGGGCAAGGATGGTTATT	ATCGCTTCCTCGTCATAGATTG
hCTSL	GGAATTGCCTCAGCTACTCTAA	CTCTCCTCCATCCTTCTTCATTC
hCTSK	GCTTCTCTTGGTGTCCATACA	GGGTACTTTGAGTCCAGTCATC
hCTSV	TGGACTCTGAGGAATCCTATCC	ACTGTGAAGCCAGTGTCATTAG
hCTSW	CCAGCATGGGCAGAGAAATA	GGTCCTTGATGGGTGAGATG
